# Modeling *Drosophila* sleep: fly in the sky?

**DOI:** 10.1093/sleep/zsad309

**Published:** 2023-12-09

**Authors:** Anne C Skeldon, Derk-Jan Dijk

**Affiliations:** School of Mathematics and Physics, Faculty of Engineering and Physical Sciences, University of Surrey, Guildford, UK; UK Dementia Research Institute Care Research and Technology Centre, at Imperial College London and the University of Surrey, Guildford, UK; UK Dementia Research Institute Care Research and Technology Centre, at Imperial College London and the University of Surrey, Guildford, UK; Surrey Sleep Research Centre, Faculty of Health and Medical Sciences, University of Surrey, Guildford, UK

The notion that sleep timing is regulated by an interaction of sleep homeostasis and circadian rhythmicity is embraced by a broad section of the sleep and circadian community and the two-process publications by Borbély et al. [1] and Daan et al. [2] are highly cited.

For many years now, multiple techniques and approaches have been applied to study sleep and circadian rhythmicity in various species. Examples include: the study of the time course of EEG slow wave activity as a marker of sleep homeostasis at baseline or following wake extension (humans [3, 4], rodents [5, 6]); forced desynchrony of sleep and circadian rhythms (humans [7, 8], rats [9], mice [10], and flies [11]); effects of sleep and wakefulness on circadian clock gene expression in mice [12, 13]; activation and inhibition of sleep-promoting neurons across the 24 hours day in rhythmic and arrhythmic flies [14]; ablation of the suprachiasmatic nucleus to remove circadian rhythmicity (rodents [15], primates [16]); altering circadian rhythmicity by mutation or removal of core clock genes in mice (e.g. [12, 17].). More recently, novel molecular genetic tools have enabled the creation of circadian chimeric mice [18] or local restoration of circadian rhythmicity only in the SCN [19] and considered the resulting effects on sleep. In humans, stratification according to clock gene variants has been used to investigate genetic contributions to sleep and sleep homeostasis [20, 21]. These and many other studies have provided valuable insights into sleep homeostasis and circadian rhythmicity and their interaction. Few researchers have investigated the interaction of sleep homeostasis and circadian rhythmicity in quantitative detail by estimating parameters of the two-process model or have applied the two-process model to non-human species.

**Figure 1.**
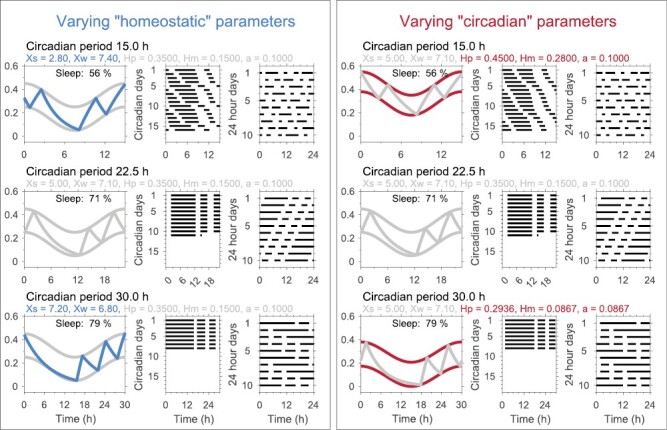
Entangled sleep and circadian rhythms in the two-process model: variation in sleep–wake phenotypes may be explained by either modifying homeostatic or circadian parameters. Here are shown sleep–wake patterns for different assumptions on which parameters vary with circadian period. Left-hand panels are for changes in the homeostatic time constants based on the trend line in Abhilash and Shafer [23] Figure 5 panels D and E (although we note that to get the impressive fit shown in Figure 5C they used individually fitted values for both the decay and buildup time constants including the large jump in homeostatic buildup constant between the circadian period of 15 and 20 hours). Right-hand panels keep homeostatic time constants fixed and vary “circadian” parameters. In each case, the two-process model is shown along with two raster plots, one based on the duration of the circadian day (left), one based on the 24-hour day (right). Like Abhilash and Shafer, all simulations use the scaled two-process model which sets the upper and lower asymptotes to 1 and 0, respectively. All other parameters are written in the figure, where Xs and Xw are the time constants for homeostatic decay during sleep and buildup during wake, respectively, Hp and Hm are the mean levels of the upper and lower thresholds and a is the circadian amplitude.

Often people consider the conceptual framework of the two-process model as a division of sleep–wake regulation into two separate processes. However, the quantitative formulation of the two-process model is fundamentally about the interaction of entangled sleep and circadian processes [22]. From a mathematical perspective, sleep and circadian parameters in the two-process model are interlinked so it is not always easy to separate out what is a “homeostatic” effect and what is a “circadian” effect (see below and Figure 1).

It is therefore with interest we see that Abhilash and Shafer [23] have constructed a two-process model for flies. Abhilash and Shafer make choices for most parameters based on Daan et al. [2] and fit the homeostatic time constants so that sleep is biphasic and the percentage of sleep across the day in the presence of a light–dark (LD) cycle (approximately 75%) matches observations. They show that ultradian rhythms present in flies without a functioning circadian system can be explained in their two-process model by the homeostatic process oscillating between two thresholds which are constant. They suggest that the time constant for the decay of homeostatic sleep pressure during sleep is larger than the time constant for the buildup of homeostatic sleep pressure during wake. They quantify the interaction of sleep homeostasis and circadian rhythmicity by estimating model parameters in strains of flies with a wide range of circadian periods (approximately 15 to 30 hours) and “reveal”—as indicated in the title of the paper—that short circadian periods are accompanied by faster rates of decay of homeostatic sleep pressure.

The authors acknowledge that, necessarily, there are some underlying assumptions in their analysis, and it is worth reflecting on the extent to which their conclusions critically depend on these assumptions.

Abhilash and Shafer found the time constants for sleep (wake) are 9.51 hours (8.76 hours) for wild-type flies studied under a 12:12 LD cycle. However, the exponential nature of sleep homeostatic means that values found for time constants fitted from patterns of sleep behavior are critically dependent on the assumed value of the circadian thresholds. A biphasic pattern with the same percentage of wake and sleep can be found for time constants for sleep (wake) as 8.76 hours (9.51 hours) i.e. exactly the reverse of those used by Abhilash and Shafer, with a very small change in the value of only the upper threshold (reducing the value from 0.35 to 0.327). Abhilash and Shafer suggest that the time constant for decay of homeostatic sleep pressure may be larger than the time constant for the buildup of homeostatic sleep pressure during wake in flies, unlike those measured in humans, because flies spend a large percentage of the 24-hour day asleep. But this cannot be deduced from the magnitude of the time constants. What can be said is that, unless there is a net increase or decrease in homeostatic sleep pressure during a circadian cycle, the average increase in homeostatic sleep pressure during wake must equal the average decrease during sleep. If longer time is spent in sleep than in wake for the same change in sleep pressure, then, on average, sleep pressure decays more slowly during sleep than it accumulates during wake. But that is geometry and requires no fitting of data to a two-process model.

Later in their paper Abhilash and Shafer fit to flies with many different circadian periods, effectively creating many different “fly models” for flies in constant darkness (DD) (see [23] Figure 5 panels D, E). Curiously, time constants for sleep (wake) of 9.51 hours (8.76 hours) are outliers and in most cases, the time constant for the decay of homeostatic sleep pressure during sleep is *smaller* than the time constant for the buildup of homeostatic sleep pressure during wake. This would imply that the presence of a LD cycle has major effects on the homeostatic parameters.

Next, in deducing that the time constant for decay of homeostasis is associated with the circadian period, Abhilash and Shafer assume that genetically modified flies have different circadian periods but that other aspects of the circadian system are unchanged. But this is also a major assumption. In Figure 1, motivated by values from [23] Figure 5, we show that changing “circadian” parameters can lead to similar changes in the statistical properties to those found by changing the homeostatic time constants. While of course, we have not done the systematic study of Abhilash and Shafer, we suspect that if they had fixed the homeostatic time constants and elected to vary other parameters, instead of finding an association between the decay of sleep homeostasis and circadian period, they would have “revealed” a link between circadian periodicity and other properties of the circadian system.

The choices that Abhilash and Shafer make raise many other questions. Why pick parameters such that their default fly model does not predict the period of ultradian sleep episodes in the absence of a functioning circadian system? What happens to total sleep time in arhythmic flies? What explains the differences between the amount and patterns of sleep under LD versus DD? Why not select parameters which better replicate the pattern of sleep (the fly model overestimates sleep at night and underestimates sleep during the day)? Why use a sinusoidal threshold if experimental data show that the circadian threshold is skewed with a maximum drive for wake at the end of the light period [11]?

In summary, Abhilash and Shafer have conducted an interesting, challenging, and unique study, which highlights the possibilities for further in-depth exploration using fly sleep as a model to probe sleep–wake regulation. When so many people use the two-process only as a conceptual framework, it is to their credit that they carry out a quantitative study and derive a *Drosophila* two-process model. But, while perhaps the beauty and a strength of the two-process model is its graphical simplicity, this graphical simplicity hides a complex heart [24, 25]. In our view, a deeper appreciation of the complicated dynamics of the two-process model is warranted before robust assertions on the relationship between sleep homeostasis and circadian rhythmicity can be made: like flies in the sky model parameters are not easy to nail down.
